# 维奈克拉、地西他滨及改良HA方案联合供者淋巴细胞输注治疗儿童造血干细胞移植后复发AML/MDS的安全性和有效性研究

**DOI:** 10.3760/cma.j.cn121090-20240317-00099

**Published:** 2024-09

**Authors:** 枫 张, 慧芳 王, 冠华 胡, 盼 锁, 露 柏, 昱 王, 晓辉 张, 晓军 黄, 翼飞 程

**Affiliations:** 北京大学人民医院，北京大学血液病研究所，国家血液系统疾病临床医学研究中心，造血干细胞移植北京市重点实验室，北京 100044 Peking University People's Hospital, Peking University Institute of Hematology, National Clinical Research Center for Hematologic Disease, Beijing Key Laboratory of Hematopoietic Stem Cell Transplantation, Beijing 100044, China

**Keywords:** 造血干细胞移植, 复发, 儿童, 白血病, 挽救性化疗, Hematopoietic stem cell transplantation, Relapse, Pediatrics, Leukemia, Salvage chemotherapy

## Abstract

**目的:**

探究急性髓系白血病（AML）/骨髓增生异常综合征（MDS）患儿异基因造血干细胞移植（allo-HSCT）后分子学复发及血液学复发者，应用维奈克拉联合地西他滨、阿糖胞苷、高三尖杉酯碱化疗及供者淋巴细胞输注（DLI）的挽救性治疗的有效性和安全性。

**方法:**

纳入2021年1月1日至2023年6月1日北京大学人民医院血液科收治的儿童AML/MDS allo-HSCT后血液学复发/分子学复发患者29例，应用维奈克拉联合地西他滨、改良HA方案进行化疗和DLI回输，评估治疗反应和不良反应。

**结果:**

所有患儿1个疗程总体反应率（ORR）为75.8％，其中血液学复发组完全缓解（CR）率88.9％（8/9），分子学复发组MRD转阴率61.1％（11/18）。3级及以上粒细胞缺乏、贫血、血小板减少发生率分别为100％、82.7％、100％。所有患儿中位粒细胞缺乏期为15 d。DLI后11.1％患儿发生Ⅲ～Ⅳ度急性移植物抗宿主病（GVHD），7.4％的患儿发生中度及以上慢性GVHD。单因素分析中，allo-HSCT时单个核细胞（MNC）回输量<10×10^8^/kg及100 d内复发对ORR有显著影响。所有患儿中位随访406 d，1年总生存（OS）率为65％，其中有治疗反应组和无治疗反应组1年OS率分别为71％和57％，差异无统计学意义（*P*＝0.164）。

**结论:**

以地西他滨、维奈克拉、高三尖杉酯碱及阿糖胞苷为基础的化疗方案联合DLI在儿童AML/MDS allo-HSCT后复发的挽救性治疗中显示出较好的反应，但达到缓解后应尽快桥接移植以获得长期生存。

## Abstract (en)

**Objective:**

To investigate the efficacy and safety of venetoclax combined with the decitabine, cytarabine, and homoharringtonine（HHT）regimen and donor lymphocyte infusion（DLI）for the preventive and salvage therapy of pediatric acute myeloid leukemia（AML）/myelodysplastic syndrome（MDS）after allogeneic hematopoietic stem cell transplantation（HSCT）.

**Methods:**

A total of 29 relapsed pediatric/minimal residual disease-positive AML after HSCT were recruited at the Peking University Institute of Hematology from January 1, 2021, to June 1, 2023. They were treated with the above combination regimen and administered with DLI after 24–48 hours at the end of chemotherapy, and the treatment response and adverse reactions were regularly assessed.

**Results:**

The overall response rate（ORR）was 75.8％, CR rate was 88.9％（8/9）in the hematologic relapse group, and MRD negativity rate was 61.1％（11/18）in the MRD-positive group. The incidence of agranulocytosis, anemia, and thrombocytopenia with a classification above grade 3 were 100％, 82.7％, and 100％, respectively. The median time of the granulocyte deficiency period was 15 days. Acute graft-versus-host diseases（aGVHD）with a classification of grades Ⅲ–Ⅳ occurred in 11.1％ of the patients after DLI, while moderate or severe cGVHD occurred in 7.4％ of the patients. The single risk factor for ORR was MNC counts of less than 10×10^8^/kg, and the relapse occurred within 100 days. At a median follow-up of 406 days, the 1-year OS was 65％, and the 1-year OS was 57％ in the group with no reaction（*P*＝0.164）compared with 71％ in the group who had an overall reaction.

**Conclusion:**

The combined regimen based on the DAC, VEN, and modified HA regimen showed a high response rate in the salvage therapy for pediatric AML after the relapse of HSCT. However, bridging to transplantation should be performed immediately after remission to result in a long survival rate.

近年来，通过强化疗联合或不联合造血干细胞移植（HSCT）儿童急性髓系白血病（AML）长期生存率可达到70％[1]。然而尽管应用HSCT，仍有30％的儿童有较高的复发风险，HSCT后复发的AML患儿，目前尚无有效的诱导治疗方案。如何令HSCT复发后儿童获得深度缓解，为后续治疗争取机会目前仍是难题。基于HSCT后的免疫状态，HSCT后复发患儿的化疗方案应满足不良反应相对弱、药物相互作用少、不加剧移植物抗宿主病（GVHD）的特点[2]–[3]。基于既往报道的AML患者应用联合方案获更深层的缓解[4]及HSCT后供者淋巴细胞输注（DLI）的有效性，本研究将维奈克拉（VEN）联合地西他滨（DAC）、改良剂量的HA及DLI方案用于HSCT后血液学或分子学复发的儿童AML的挽救性化疗，旨在评估治疗有效性和安全性，为此类患儿的挽救性治疗提供更多选择。

## 病例与方法

一、病例

纳入2021年1月1日至2023年6月1日北京大学人民医院血液科收治的行同胞全相合HSCT及半相合HSCT的儿童AML/骨髓增生异常综合征（MDS）患者，所有患儿HSCT后1、2、3、4.5、6、9、12个月复查骨髓，之后每半年复查1次，至移植后5年。监测骨髓涂片、免疫残留、嵌合情况，髓外复发以PET-CT确认。最终29例HSCT后血液学或分子学复发患儿纳入本研究。主要纳入标准：①骨髓细胞形态学提示原始细胞>5％，或免疫残留或融合基因阳性（≥0.01％）提示分子学复发，或PET-CT提示髓外复发者；②无Ⅱ度及以上急性GVHD（aGVHD）或系统性慢性GVHD（cGVHD）；③肝功能丙氯酸转氨酶≤正常值上限的2倍，天冬氨酸转氨酶≤正常值上限的2倍，总胆红素≤20 mg/L；④心脏射血分数（EF）值≥50％；⑤患儿监护人均签署知情同意书。排除标准：①存在Ⅱ度及以上aGVHD或系统性cGVHD；②肝功能丙氨酸转氨酶>正常值上限2倍或天冬氨酸转氨酶>正常值上限2倍或总胆红素>20 mg/L；③心脏EF值<50％；④各种原因放弃治疗。本项研究获得北京大学人民医院伦理委员会审批（批件号2023PHB204-001）。

二、治疗方案

1. 预处理方案：同胞全相合HSCT采用常规改良BU/CY（白消安+环磷酰胺）方案，半相合HSCT在BU/CY基础上加用抗胸腺细胞免疫球蛋白（ATG）2.5 mg/kg（−5，−4，−3，−2 d）。HSCT前伴髓外浸润者予全身放疗。HSCT前融合基因或流式细胞术（FCM）检测显示微小残留病（MRD）阳性者加予DAC 200 mg/m^2^（−11，−10 d）加强预处理。

2. 干细胞动员和采集：全部患者的供者采用G-CSF进行干细胞动员，移植物均采用外周血。

3. 急性GVHD预防：HSCT后采用环孢素A（CsA）+吗替麦考酚酯（MMF）+短疗程甲氨蝶呤（MTX）方案预防GVHD。复发后DLI前24 h再次加用CsA，目标浓度150～200 µg/L，回输6周后开始减量，8周减停，如有GVHD则延长CsA应用时间。

4. 复发后治疗方案：VEN 100 mg/m^2^第1天至第14～21天［用药第7天监测VEN浓度，目标谷浓度1 000 µg/L，服药后6 h浓度2 000 µg/L，据血药浓度以25 mg增减至目标范围，同时预防量口服唑类抗真菌药以提高VEN血药浓度，出现粒细胞缺乏（粒缺）伴发热停用］；DAC 20 mg/m^2^，第1天至第5～7天；高三尖杉酯碱（HHT）1 mg/m^2^，第1天至第5～7天；阿糖胞苷（Ara-C）100 mg/m^2^，第1天至第5～7天。化疗结束后24～48 h予DLI，回输单个核细胞（MNC）为1×10^8^/kg。粒缺期予G-CSF刺激造血，红细胞、血小板输注等支持治疗。

三、疗效评价与随访

完全缓解（CR）：骨髓无白血病状态伴中性粒细胞绝对计数（ANC）和PLT完全恢复。完全缓解伴不完全血细胞恢复（CRi）：骨髓无白血病状态伴ANC、PLT未完全恢复。部分缓解（PR）：骨髓原始细胞>5％且≤20％，白血病细胞下降>50％。总体反应率（ORR）：CR率、CRi率及PR率之和。未缓解（NR）：未能获得CR、CRi或PR。不良事件参考美国CTCAE第5版，主要评估血液学和非血液学不良反应。GVHD分度参考《中国异基因造血干细胞移植治疗血液系统疾病专家共识（Ⅲ）——急性移植物抗宿主病（2020年版）》[5]。所有患儿化疗后骨髓抑制期每日进行血常规、生化检查至血细胞恢复。自DLI回输之日起+1个月进行骨髓检查评估治疗反应，+2个月第2次复查，根据骨髓情况及患者监护人意愿选择巩固化疗、二次移植或密切随访。所有患儿均规律行骨髓穿刺检查，定期门诊或电话随访。末次随访日期2024年2月1日，中位随访时间406 d。

四、统计学处理

应用SPSS 26.0软件进行统计分析，计数资料采用频数和百分率（％）表示，组间比较采用卡方检验或Fisher确切概率法。计量资料采用Mann-Whitney *U*检验，对影响ORR的因素进行单因素分析。*P*<0.05为差异有统计意义。

## 结果

一、一般资料

共纳入29例患者，其中男17例，女12例，中位发病年龄6（1～14）岁，原发病为AML 27例，其中低危6例，中危8例，高危13例；MDS 2例。按FAB分型AML-M_2_ 13例，AML-M_5_ 7例，AML-M_7_ 2例，5例未明确具体分型。诊断至HSCT的中位时间为160（63～1 144）d，4例行同胞全相合HSCT，余25例为半相合HSCT。23例CR_1_行HSCT，5例CR_2_行HSCT，1例MDS-IB期直接行HSCT。8例加用DAC强化处理，其余为常规预处理方案。所有患儿中位回输MNC 10.45（7.49～14.33）×10^8^/kg，中位回输CD34^+^细胞6.295（1.99～17.64）×10^6^/kg。所有患儿粒系均植活，中位植活时间12（10～25）d。除1例血小板植入不良，其余血小板均植活，中位植活时间12（8～43）d，1例出现继发植入不良。HSCT后至复发前31.0％患儿出现Ⅱ～Ⅳ度aGVHD，10.3％的患儿出现Ⅲ～Ⅳ度aGVHD，未观察到系统性cGVHD（表1）。

**表1:** 29例HSCT后复发AML/MDS患儿基线资料

临床特征	例数	百分比（%）
性别		
男	17	58.6
女	12	41.4
年龄		
<10岁	20	69.0
≥10岁	9	31.0
疾病起源		
原发AML	25	86.2
tAML/MDS	2	6.9
MDS	1	3.4
MDS转化型AML	1	3.4
FAB分型		
AML-M_2_	13	44.8
AML-M_5_	7	24.1
AML-M_7_	2	6.9
MDS	2	6.9
未分类	5	17.2
融合基因		
ETO::AML1	6	20.7
KMT2A-r	4	13.8
DEK::NUP214	1	3.4
NUP98-r	2	6.9
TLS::ERG	3	10.3
危险度		
低危	6	22.2
中危	8	29.6
高危	13	48.1
HSCT前疾病状态		
CR_1_	23	79.3
CR_2_	5	17.2
PR	1	3.4
MRD		
阳性	17	58.6
阴性	12	41.4
预处理(半相合)		
BU/CY/ATG	15	51.7
DAC+BU/CY/ATG	6	20.7
TBI/CY/ATG	1	3.4
预处理（全相合）		
BU/CY	2	6.9
DAC+BU/CY	2	6.9
供受者血型		
主要不合	5	17.2
次要不合	3	10.3
相合	21	72.4
DAC暴露史		
有	10	34.5
无	19	65.5
GVHD		
Ⅱ~Ⅳ度aGVHD	9	31.0
Ⅲ~Ⅳ度aGVHD	3	10.3
系统性cGVHD	0	0
HSCT后复发情况		
分子学复发	18	62.0
血液学复发^a^	9	31.0
单独髓外复发	2	7.0
嵌合情况		
供者嵌合	19	65.5
混合嵌合	10	34.5

**注** HSCT：造血干细胞移植；AML：急性髓系白血病；tAML：治疗相关AML；MDS：骨髓增生异常综合征；CR_1_：第1次完全缓解；PR：部分缓解；MRD：微小残留病；DAC：地西他滨；GVHD：移植物抗宿主病；aGVHD：急性GVHD；cGVHD：慢性GVHD；BU/CY/ATG：白消安+环磷酰胺+兔源抗胸腺细胞球蛋白；TBI：全身放疗；^a^包括伴髓外复发者

二、复发情况

29例患儿中分子学复发18例，血液学复发伴或不伴髓外复发9例，单独髓外复发2例，所有患儿中位复发时间为移植后272（56～685）d。其中HSCT至分子学复发的中位时间为233（78～638）d，HSCT至血液学复发的中位时间为279（56～685）d，两组复发时间差异无统计学意义。两组患儿均接受相同化疗方案，具体天数视骨髓抑制情况而定。

三、疗效

18例分子学复发组患者11例MRD转阴，转阴率为61.1％，2例MRD较前下降50％以上，ORR为72.2％。9例血液学复发组患者1例NR，其余均获得CR，总体CR率88.9％；其中MRD完全转阴6例（66.7％）。2例髓外复发者1个疗程均评估PET-CT，1例CR，1例PD。所有患儿ORR为75.8％。将年龄、性别、原发病类型、移植前疾病状态、是否有DAC暴露史、复发时间、复发类型、是否存在GVHD等因素纳入影响ORR的单因素分析，发现移植时回输MNC数量<10×10^8^/kg、复发时间<100 d对ORR具有显著影响（*P*<0.05）（表2）。

**表2:** 影响造血干细胞移植后复发AML/MDS患儿化疗联合供者淋巴细胞输注ORR的单因素分析

因素	例数	ORR（%）	*χ*^2^值	*P*值
性别			0.624	0.430
男	17	70		
女	12	83		
年龄			0.026	0.872
≥10岁	9	77		
<10岁	20	75		
DAC暴露			2.097	0.148
有	10	60		
无	19	84		
Ⅲ~Ⅳ度GVHD			3.305	0.069
有	3	33		
无	26	89		
移植MNC数量			4.340	0.037
≥10×10^8^/kg	14	93		
<10×10^8^/kg	15	60		
复发时间			5.489	0.019
<100 d	7	43		
≥100 d	22	86		
危险度			1.952	0.229
低危	6	100		
中危	8	77		
高危	13	64		
MRD			0.343	0.558
阳性	17	72		
阴性	12	81		
移植时状态			0.830	0.362
CR_1_	23	79		
CR_2_	5	60		
移植类型			1.695	0.193
半相合	25	80		
全相合	4	50		
复发			1.695	0.429
分子学	18	72		
血液学	9	88		
髓外	2	50		

**注** AML：急性髓系白血病；MDS：骨髓增生异常综合征；ORR：总反应率；DAC：地西他滨；GVHD：移植物抗宿主病；MNC：单个核细胞；MRD：微小残留病；CR_1_：第1次完全缓解

四、不良反应

所有患儿均经历了粒缺期，中位粒缺时间15（8～47）d。血液学不良反应中，所有患儿均出现了3级及以上粒缺及血小板减少，其余严重非血液学不良反应包括黏膜炎、脓毒症、重症肺炎（表3）。所有患儿化疗后均存活，无治疗相关死亡。除2例因严重感染未回输，共27例患儿进行DLI，5例患儿发生Ⅱ度GVHD，3例患儿发生Ⅲ～Ⅳ度GVHD，2例患儿发生系统性cGVHD。

**表3:** 造血干细胞移植后复发AML/MDS患儿化疗联合DLI的不良反应

不良反应（≥3级）	例数	百分比（%）
血液学不良反应		
粒缺伴发热	18	62.1
粒细胞缺乏	29	100
贫血	24	82.7
血小板减少	29	100
非血液不良反应		
口腔黏膜炎	3	10.0
肺炎	6	20.7
胆红素升高	1	3.4
丙氨酸转氨酶升高	5	17.2
天冬氨酸转氨酶升高	5	17.2
低钾血症	2	6.9

**注** AML：急性髓系白血病；MDS：骨髓增生异常综合征；DLI：供者淋巴细胞输注

五、后续监测及随访

所有患儿中，7例治疗1个疗程后行二次移植，其中6例为血液学缓解状态移植，存活5例，1例复发死亡；1例NR状态于外院行HSCT，复发死亡。余22例未直接行HSCT患者中，5例血液学复发，7例MRD转阳，3例中枢神经系统复发；其中5例经靶向治疗/DLI联合化疗后血液学缓解状态下行二次移植，除1例死于脓毒症，其余4例均存活。17例患儿未选择HSCT，7例死亡，2例带瘤存活，余9例截至末次随访，仍处于无病生存状态。所有患儿中位随访406 d，1年OS率为65％，获得治疗反应组与无治疗反应组1年OS率分别为71％和57％，差异无统计学意义（*P*＝0.164）（图1）。

**图1:**
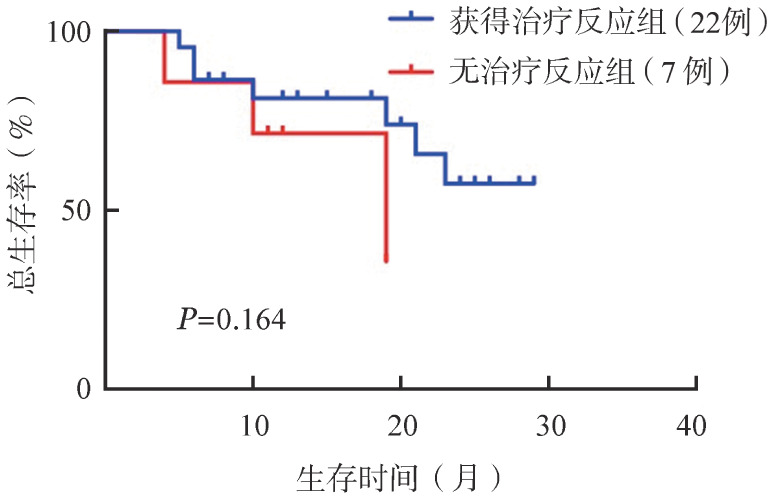
造血干细胞移植后复发患儿化疗联合供者淋巴细胞输注获得治疗反应组与无治疗反应组生存情况比较

## 讨论

HSCT后复发患儿对常规化疗不敏感，脏器功能更差，且混合有GVHD表现，选择标准方案治疗风险更大且获益更低[6]。一项大鼠的移植模型显示VEN通过抑制移植前NK细胞活性，进而提高移植活率，并不加重GVHD作用[7]，提示联合方案用于移植后化疗的可能性。本研究中，联合疗效案在移植后复发AML/MDS儿童中展示出良好的疗效，血液学复发组总体CR率达88.9％，其中66.7％的患儿MRD完全转阴；分子学复发组MRD转阴率61.1％，ORR为72.2％；两组患儿的ORR达75.8％。

去甲基化药物（HMA）被广泛应用于un-fit AML的诱导治疗和常规诱导治疗失败的AML[8]–[9]，其应用于移植后复发AML患儿的挽救性治疗鲜有报道，相较于阿扎胞苷（AZA）用于HSCT后的预防及挽救性治疗[3]，应用DAC作为复发后挽救性治疗的报道更少，且多以成人患者为主[10]。美国MD Anderson癌症中心[11]报道DAC及VEN在难治/复发（R/R）AML中展现出更高的反应率和更低的药物毒性。一项来自德国的多中心研究报道DAC联合DLI可用于allo-HSCT后复发的挽救性治疗，也可作为AZA失败后的第二选择[12]。VEN联合HMA治疗R/R AML的CR率为44％～59％，并展现出同标准诱导化疗相近的疗效[13]–[14]。既往有研究认为VEN+AZA+HHT的联合方案相比VEN联合HMA CR率更高（63.8％对40.9％）[8]。在一项纳入20例包括老年及儿童R/R AML的单中心研究中，VEN+DAC+CAG（阿糖胞苷+阿柔比星+G-CSF）的联合方案获得90％的ORR，CR率高达85％[15]。

国内陈欣等[16]报道成人HSCT后复发患者单独应用VEN及DLI回输的治疗反应，分子学复发组ORR为80％，血液学复发组的ORR为60％，本研究ORR高于陈欣等的报道，可能与加用HMA，且大部分患儿行DLI回输，增加了化疗作用有关。最近一项纳入10项研究、243例成人患者的meta分析显示，HSCT后复发患者应用VEN联合HMA治疗的CR率为32％，ORR为48％，6个月OS率为42％，1年OS率为23％[17]。相较于其他中心方案，我中心应用DAC联合HA方案，提高了CR率和OS率，且儿童患者合并症少，耐受程度优于成人患者，这可能是本中心ORR高于既往报道的原因。本研究发现，移植时MNC<10^8^/kg者，挽救化疗的ORR更低，可能与MNC数量高，提高了GVHD及移植物抗白血病（GVL）效应有关。

联合治疗更高的ORR也带来更多的不良事件，本研究中Ⅲ级以上血液学不良事件发生率高，粒细胞缺乏、贫血、血小板减少发生率分别为100％、82％、100％。而文献报道单独DAC用于诱导治疗和HSCT后预防复发时Ⅲ级以上血液学不良事件的发生率为20％～45％，尽管粒缺发热、肺炎等感染的发生率大致相同[18]–[19]。提示联合方案的粒缺期更长，需更积极地处理并发症，避免治疗相关死亡发生。

我中心既往研究认为应用DAC后缓解患者的T细胞恢复较未缓解者更快，提示DAC和DLI具有协同作用，在增加GVL效应的同时并不增加GVHD风险，可能与DAC降低肿瘤负荷，有利于调节性T细胞功能恢复，进而降低了GVHD风险有关[20]。本研究中，5例存在Ⅱ～Ⅳ度GVHD，2例存在Ⅲ～Ⅳ度GVHD，发生率低于既往单独应用DLI者[21]。本研究中是否存在Ⅲ～Ⅳ度aGVHD及cGVHD并未增加ORR，也验证了DAC在抗白血病治疗同时有助于T细胞恢复，故并未增加GVHD发生率。

本方案1个疗程后可获得较高的CR率，为此类患者提供了后续治疗的机会，获得治疗反应组的1年OS率明显高于无反应组。本研究中7例患者1个疗程后桥接移植，CR后的长期OS率明显提高。1个疗程后未行二次移植的患者，后续再次化疗和（或）靶向治疗最终复发率为75％。提示作为挽救性治疗，DAC为复发患者获得了缓解并争取了后续移植的机会，但仅靠化疗并不能令患儿长期获益，仍需更积极的治疗使此类患儿获得长期生存。

本研究存在一定局限性，纳入例数较少，且为单臂研究，后续仍需进行随机对照研究，以评估其和常规化疗相比的安全性和有效性。
